# Initial and advanced endoscopic findings of monomorphic epitheliotropic intestinal T‐cell lymphoma in the duodenum: A case report

**DOI:** 10.1002/deo2.118

**Published:** 2022-04-01

**Authors:** Masanori Fukushima, Tetsuro Honda, Naohiro Komatsu, Ryu Sasaki, Eisuke Ozawa, Satoshi Miuma, Hisamitsu Miyaaki, Junji Irie, Shinji Okano, Kazuhiko Nakao

**Affiliations:** ^1^ Department of Gastroenterology and Hepatology Nagasaki University Graduate School of Biomedical Sciences Nagasaki Japan; ^2^ Department of Gastroenterology and Hepatology Nagasaki Harbor Medical Center Nagasaki Japan; ^3^ Department of Pathology Nagasaki Harbor Medical Center Nagasaki Japan; ^4^ Department of Pathology Nagasaki University Graduate School of Biomedical Sciences Nagasaki Japan

**Keywords:** duodenum, endoscopic findings, ground cracking, microgranular pattern, monomorphic epitheliotropic intestinal T‐cell lymphoma

## Abstract

Monomorphic epitheliotropic intestinal T‐cell lymphoma (MEITL) is an aggressive malignant digestive system lymphoma. We report the case of a 68‐year‐old Asian woman who was diagnosed with MEITL of the duodenum and small intestine due to intestinal obstruction. MEITL is mainly located in the small intestine, and duodenal lesions are rare. Therefore, the endoscopic appearance of MEITL in the duodenum has been reported in only a few cases. In this case, we observed the initial and advanced endoscopic findings of MEITL in the duodenum. The initial findings were only slight mucosal changes; therefore, careful observation is required to detect early‐stage MEITL.

## INTRODUCTION

Monomorphic epitheliotropic intestinal T‐cell lymphoma (MEITL) has been defined as a new disease, instead of type 2 enteropathy‐associated T‐cell lymphoma, as per 2017 WHO classification.^1^

MEITL is a rare disease with a very poor prognosis, mainly located in the small intestine, particularly the jejunum.^2^ More than half of the patients present acutely with intestinal perforation or obstruction; therefore, a few reports of the endoscopic findings of MEITL are available. Herein, we present a case of MEITL, where we were able to observe the initial and advanced endoscopic findings of the duodenum over time. We believe that our case may provide a better understanding of this rare disease and improve patient care.

## CASE REPORT

**FIGURE 1:**
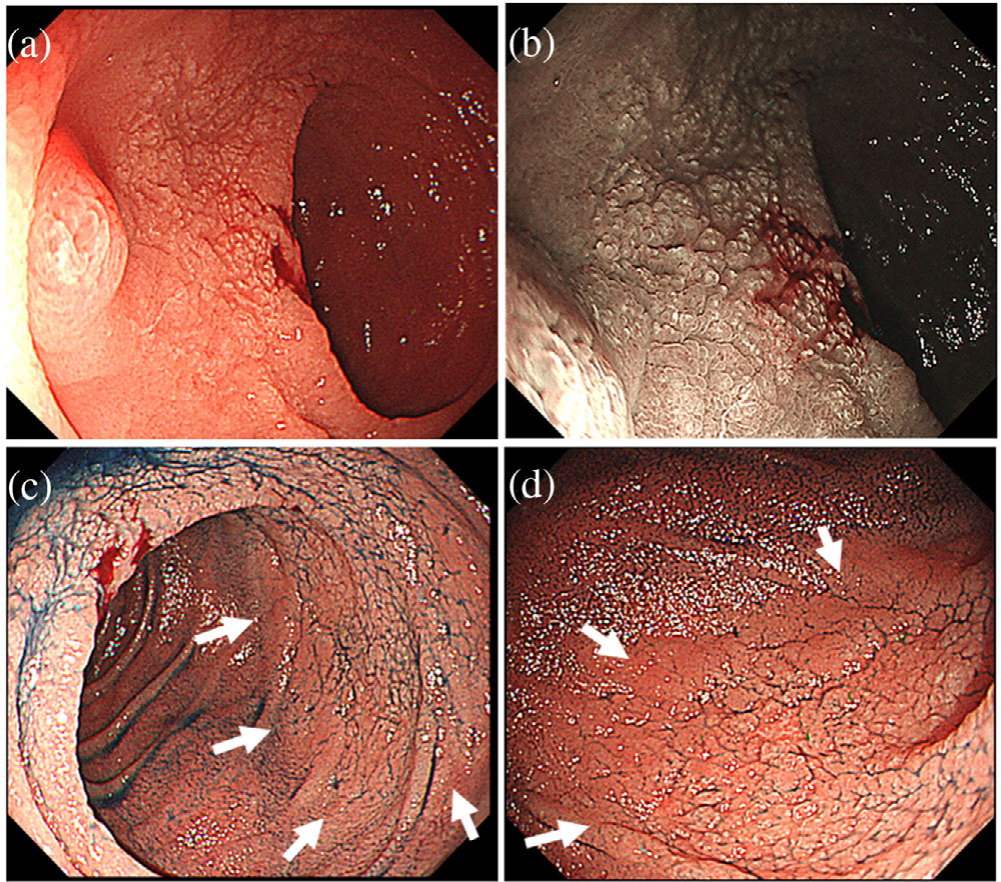
(a) The second portion of the duodenum shows the microgranular mucosa with white villi. (b) Narrow‐band imaging shows the microgranular mucosa clearly. (c,d) The boundaries of the lesions (white arrows) are relatively clear beside the slight depression, and show a mucosal pattern, like ground cracking, on the borderline (indigo carmine spray enhancement)

**FIGURE 2:**
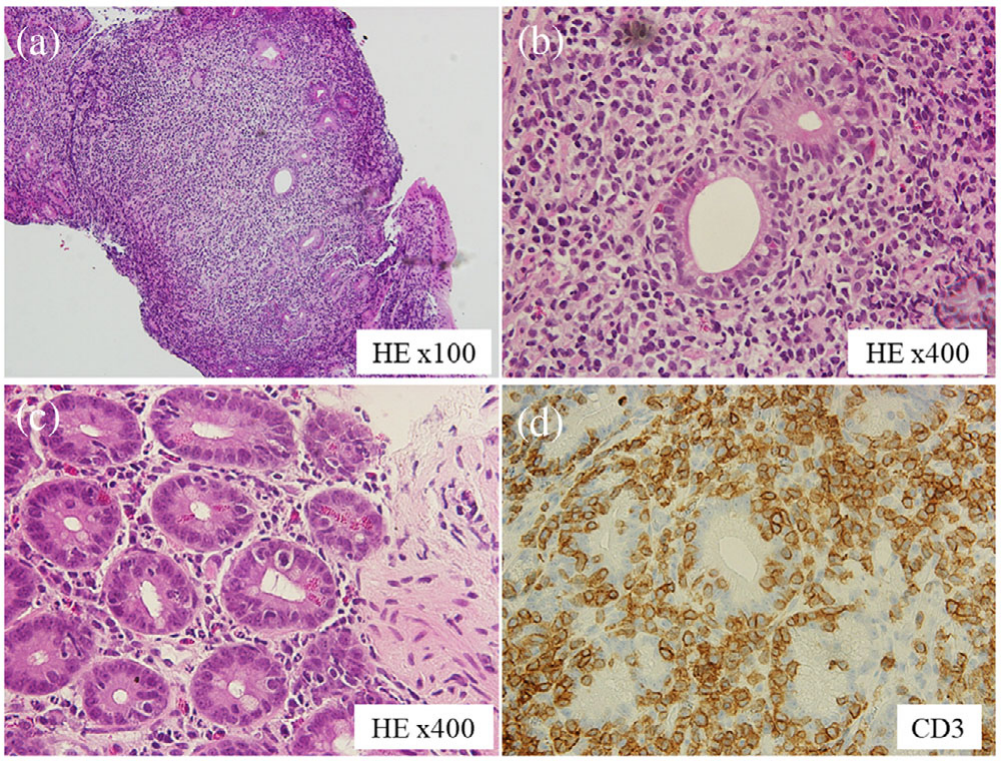
(a–c) A diffuse infiltrate of small to medium size atypical lymphocytes and atypical intraepithelial lymphocytes are seen in the duodenum. (d) Immunohistochemistry analysis shows that tissue biopsy has CD3‐positive intraepithelial lymphocytes

**FIGURE 3:**
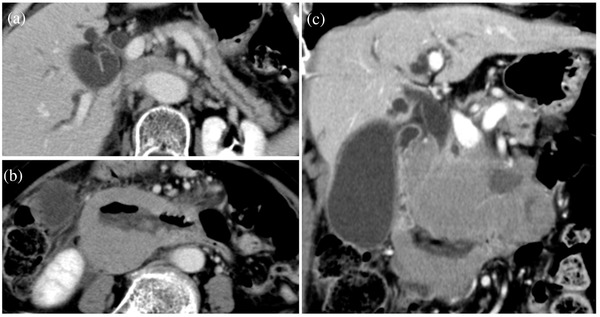
Computed tomography images: Abdominal computed tomography images of transverse sections (a,b) and coronal section (c) reveal a mass in the second portion of the duodenum. Duodenal stenosis, pancreatic duct dilatation, and bile duct obstruction are present

**FIGURE 4:**
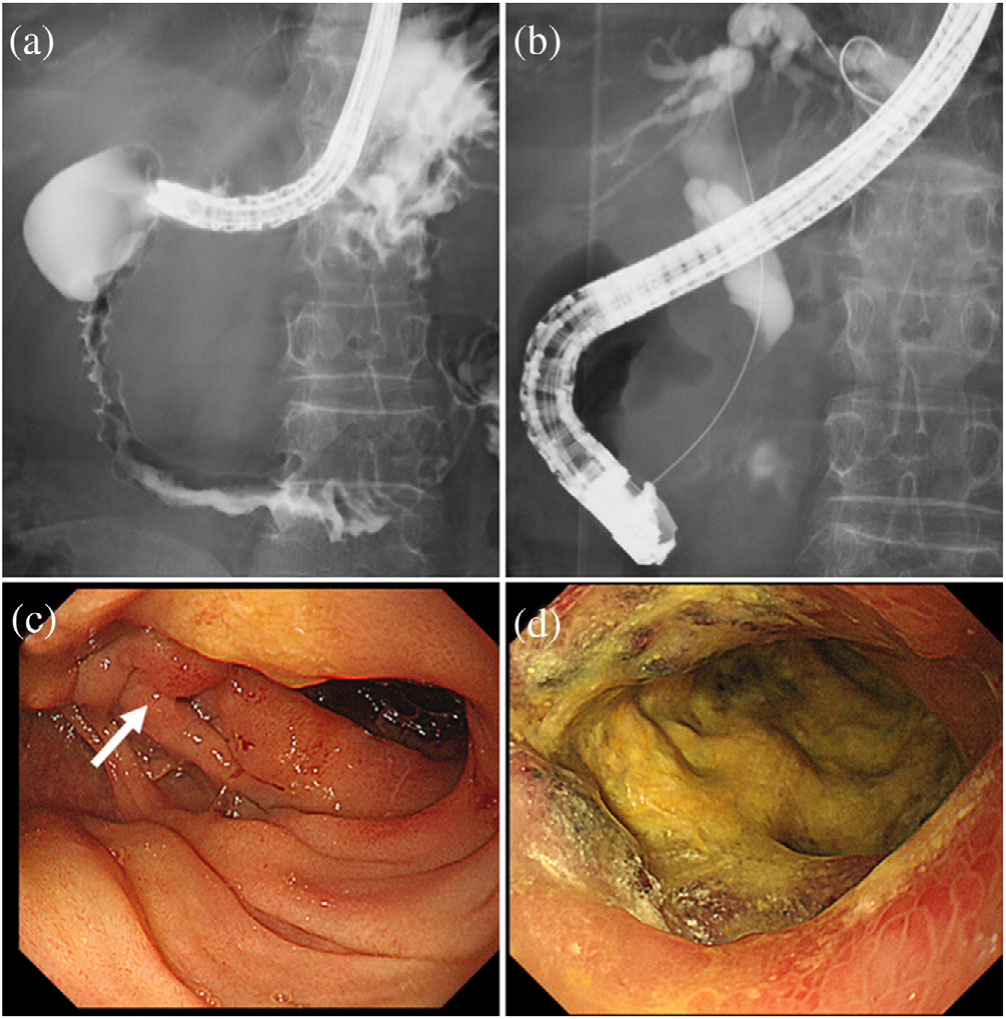
(a) Upper gastrointestinal series shows stenosis extending to the second and third portions of the duodenum. (b) Endoscopic retrograde biliary drainage is performed. (c,d) An upper endoscopy shows a circumferential ulcer and stenosis in the second and third portions of the duodenum. The papilla of Vater remains on the margin of the circumferential ulcer (white arrow)

A 68‐year‐old Asian woman presented with upper abdominal pain and nausea. Computed tomography (CT) revealed small intestinal obstruction, and she was treated conservatively. However, due to persistent upper abdominal pain, upper endoscopy was performed, which revealed relatively well‐defined microgranular changes from the duodenal papilla to the horizontal region (Figure 1). The stomach appeared normal. A biopsy of the microgranular mucosa of the duodenum showed proliferation of small‐ or medium‐sized atypical T‐cell lymphocytes and intraepithelial lymphocytes (Figure 2). Immunohistochemistry analysis showed that tissue biopsy was positive for CD3, CD8, CD56, and granzyme B, and negative for CD4, CD20, and CD30. The laboratory tests revealed a soluble interleukin 2 receptor (sIL2R) level of 797 U/ml (normal range: 121–613 U/ml). Based on these results, the patient was diagnosed with MEITL. The small intestinal obstruction did not improve with conservative treatment; hence, laparoscopic partial small intestinal resection was performed for diagnostic and therapeutic purposes. The resected specimen showed stenosis with ulceration, and additionally, the diagnosis of MEITL (Figure S1). Positron emission tomography (PET)/CT revealed high ^18^F‐fluorodeoxyglucose (^18^F‐FDG) accumulation in another part of the small intestine in the pelvic cavity and in the mesenteric lymph nodes. There were no findings in bone marrow aspiration. The patient was diagnosed as MEITL in Lugano stage Ⅱ. Therefore, auto‐peripheral blood stem cell transplantation (PBSCT) was performed in the department of hematology to obtain a complete response. Twenty‐two months had passed since diagnosis, without a relapse. In the 23rd month, the patient presented with jaundice and abdominal pain. Although no lesion had been identified by CT 3 months ago and sIL2R was within normal levels in the laboratory test 1 month ago, contrast‐enhanced CT revealed a huge extramural tumor of the duodenum with a diameter of 7 cm, and the sIL2R level was elevated at 1822 U/ml. The duodenum and the bile duct were obstructed by the tumor (Figure 3). Upper endoscopy showed a circumferential ulcer and stenosis, in the second and third portions of the duodenum (Figure 4). The papilla of Vater remained on the margin of the circumferential ulcer; therefore, we performed endoscopic retrograde biliary drainage. Histological examination of the duodenal biopsies showed MEITL recurrence. The patient received chemotherapy; however, she died of infection 1 month later.

## DISCUSSION

We report a case of MEITL, in which we observed the initial and advanced endoscopic findings in the duodenum over time. MEITL develops in the small intestine and is often diagnosed at the time of intestinal obstruction or perforation. Therefore, there are a few reports of the endoscopic findings of MEITL in the literature.

MEITL is defined as a primary intestinal T‐cell lymphoma derived from intraepithelial lymphocytes, characterized by monotonous cytomorphology and infiltration of the intestinal epithelium, without a clear association with coeliac disease.^3^ MEITL occurs predominantly in Asia and is less common in Western populations.^4^ The most common site of MEITL is the small intestine, accounting for 90% of cases, hence it is rarely found in the duodenum.^4^ Lymphoma cells are positive for CD3, CD8, and CD56 and negative for CD30.^1^

At present, there are no standardized treatment protocols for combination chemotherapy for MEITL. However, patients who underwent high‐dose chemotherapy with autologous hematopoietic stem cell transplantation (HSCT) displayed better survival rates than those who received anthracycline‐based therapies.^5^ Therefore, HSCT should be considered a suitable consolidation therapy once an initial response is obtained, particularly when autologous HSCT is applicable to many patients with the absence of marrow infiltration. In our case, auto‐PBSCT resulted in long‐term remission.

The prognosis of MEITL is generally poor. A previous study reported the median progression‐free survival of patients with MEITL to be 3 months, and the median overall survival was 7 months.^5^ The reasons for the poor prognosis of MEITL are that the clinical course is very aggressive and early detection is difficult, due to the small intestinal lesions. In the present case, although there were no abnormal findings on abdominal CT 3 months before, jaundice had appeared, indicating a recurrence.

Early diagnosis of MEITL may contribute to improving progression. Therefore, it is important to consider the possibility of MEITL based on the radiological and endoscopic findings.

MEITL is mostly localized in the small intestine, however, it includes duplications; 16% of cases are in the large intestine, 10% in the duodenum, and 5% in the stomach.^4^ Therefore, some cases can be diagnosed using upper endoscopy or colonoscopy. Initial endoscopic findings of MEITL are necessary for early detection. However, though a variety of endoscopic findings of MEITL have been reported, only a few published reports are available regarding the initial endoscopic findings. The endoscopic findings reported thus far include, diffuse thickening of the mucosa or edematous mucosa, diffuse fine granularity, multiple discrete ulcers, punched‐out ulcers, and shallow circumferential ulcers.^6^, ^7^, ^8^ Duodenal lesions of MEITL that showed edematous and granular mucosa with or without villous atrophy have been reported earlier as the initial endoscopic findings.^9^ In our case, during the first observation, the duodenum showed microgranular mucosa with white villi. While these findings were like those reported earlier, they did not show edematous changes. Additionally, in our case, the boundaries of the lesions were relatively clear beside the microgranular mucosa with a slight depression, and showed a mucosal pattern, like ground cracking, on the borderline. A biopsy of the microgranular mucosa with the slight depression, in the duodenum, yielded a diagnosis of MEITL, and an accurate diagnosis was obtained prior to resection of the small intestinal lesion. This duodenal lesion had no wall thickening on CT, no accumulation of 18F‐FDG, and could not be identified, except by endoscopy. Therefore, we suggest that these endoscopic findings of microgranular mucosa with slight depression and white villi can also be considered as the initial findings of MEITL in the duodenum.

At the time of recurrence, endoscopic findings of the duodenum showed a circumferential ulcer and stenosis, in the advanced lesion. Although PET/CT and CT were performed for follow‐up after remission, we should have additionally performed endoscopy at regular intervals, for the early detection of recurrence. Endoscopic retrograde biliary drainage was performed to clear the obstruction of the bile duct. The procedure was successful, and there were no complications. However, percutaneous transhepatic biliary drainage or endoscopic ultrasonography‐guided hepaticogastrostomy may be recommended for the treatment of obstructive jaundice caused by MEITL of the duodenum, since MEITL is prone to perforation due to generalized destructive growth without connective tissue and are extremely fragile tumors.^10^

In conclusion, we have described a case of MEITL, in which we observed the initial and advanced endoscopic findings of the duodenum. Early diagnosis of MEITL may lead to an improved prognosis; therefore, we should pay particular attention to the slight mucosal changes in the duodenum. Additionally, in cases where MEITL is in remission, endoscopic follow‐up is necessary for the early detection of recurrence, along with PET/CT or CT.

## CONFLICT OF INTEREST

None.

## FUNDING INFORMATION

None.

## Supporting information




**Supporting Information Figure S1**: Histological and immunohistochemistry findings obtained from the small intestine surgical specimen. (a,b): There is lymphocytic infiltration and proliferation in the mucosal epithelium and submucosa. Lymphocytes are monomorphic and medium‐sized. (c–h): Immunohistochemistry analysis is positive for CD3, CD8, and CD56, and negative for CD4, CD30, and CD5.
