# Supplementary figures and images for: Initial and advanced endoscopic findings of monomorphic epitheliotropic intestinal T‐cell lymphoma in the duodenum: A case report
Source: DEN Open. 2022 Apr 1;2(1):e118. doi: 10.1002/deo2.118 (PMC9302330; doi:10.1002/deo2.118)

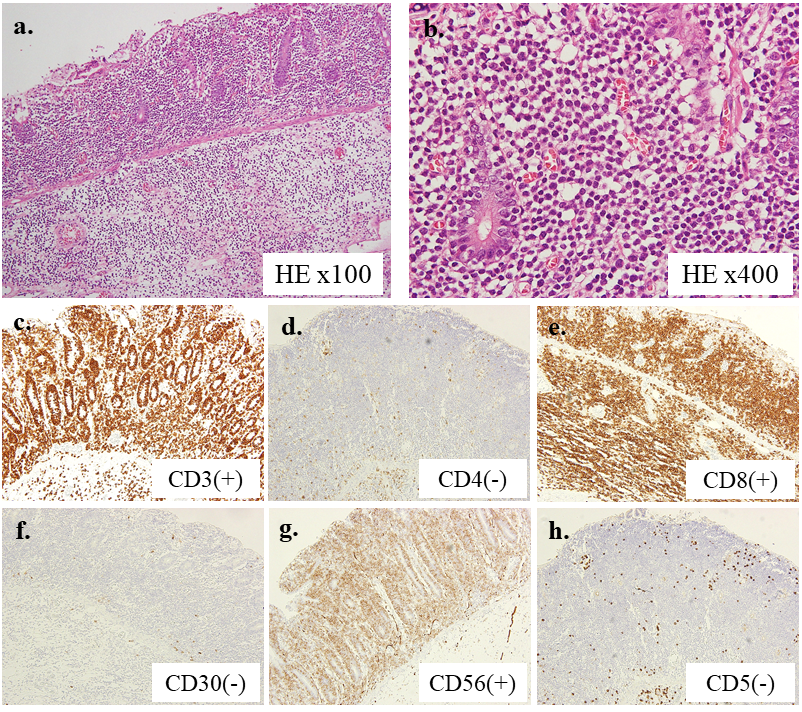

Supplement: Supplementary file 1 — Supporting Information Figure S1: Histological and immunohistochemistry findings obtained from the small intestine surgical specimen. (a,b): There is lymphocytic infiltration and proliferation in the mucosal epithelium and submucosa. Lymphocytes are monomorphic and medium‐sized. (c–h): Immunohistochemistry analysis is positive for CD3, CD8, and CD56, and negative for CD4, CD30, and CD5. [file DEO2-2-e118-s001.tif]
